# Prevalence Ratio of Otitis Media with Effusion in Laryngopharyngeal Reflux

**DOI:** 10.1155/2019/7460891

**Published:** 2019-01-01

**Authors:** Mahastini Karyanta, Siswanto Satrowiyoto, Dian Paramita Wulandari

**Affiliations:** Faculty of Medicine, Public Health and Nursing Gadjah Mada University/Sardjito General Hospital, Yogyakarta, Indonesia

## Abstract

**Background:**

Otitis media with effusion (OME) in adults is less prevalent than in the pediatric population but still causes considerable morbidity. It has been suggested that laryngopharyngeal reflux (LPR) may have a role in the aetiology of adult OME. Reflux advances to the laryngopharynx and, subsequently, to other regions of the head and neck such as oral cavity, nasopharynx, nasal cavity, paranasal sinuses, and even middle ear with clinical manifestations being asthma, sinusitis, and otitis media.

**Objective:**

To determine the prevalence ratio of otitis media with effusion in laryngopharyngeal reflux.

**Methods:**

Observational analytic with cross sectional design.

**Result:**

9 of 28 subjects experienced OME in LPR group, and 2 of 28 subjects in non-LPR group. Statistically there was significant difference between the two groups with p-value 0.02 and with 95% confidence interval range of 1.066-18.990.

**Conclusion:**

The prevalence ratio of otitis media with effusion in laryngopharyngeal reflux group is 4.5 times that in non-laryngopharyngeal reflux group.

## 1. Introduction

Otitis media with effusion (OME) is a common condition in the pediatric population. It is associated with many factors, including adenoidal hypertrophy, upper respiratory tract infection, cleft palate, and exposure to cigarette smoke. In adults, OME is less prevalent, but still causes considerable morbidity. While adult OME was once a neglected subject in terms of research effort, this is no longer the case. Over the last 20 years, a great deal of new information that sheds some light on the pathogenesis of this enigmatic condition has become available [1, 2].

The possible aetiologies and risk factor of adult OME are local malignancy, sinonasal disease, gastroesophageal reflux, eustachian tube dysfunction, smoking, intensive care patients, human immunodeficiency virus (HIV), and sarcoidosis [1]. Other common cause is allergy, which was reported in 41,9% of cases [3].

While gastroesophageal reflux disease (GERD) has long been identified as a source of esophageal disease, laryngopharyngeal reflux (LPR) has only recently been implicated in causing head and neck problems. Reflux that advances to the laryngopharynx and, subsequently, to other regions of the head and neck such as the larynx, oral cavity, nasopharynx, nasal cavity, paranasal sinuses, and even middle ear could cause LPR [4]. The most common manifestation of LPR is reflux laryngitis. Other manifestations in the head and neck that have been reported include asthma, sinusitis, and otitis media [5]. This study aimed to determine the prevalence ratio of otitis media with effusion in laryngopharyngeal reflux.

## 2. Study Method

This study was an observational analytic study with cross sectional design to determine the prevalence ratio of otitis media with effusion in laryngopharyngeal reflux. Samples are patients with throat complaints that come to the outpatient unit of Otorhinolaryngology-Head and Neck Surgery Department, Dr. Sardjito General Hospital, from October to November 2016 that meet the inclusion and exclusion criteria. The inclusion criteria for the sample in this study were (1) patients with throat complaints (hoarseness, a sense of a lump in the throat, sore throat, cough, feeling that there is mucus in the throat, difficulty in swallowing, and difficulty in breathing or choking); (2) age over 18 years; (3) being willing and able to follow the study procedures. Exclusion criteria in this study were (1) patients with acute pharyngitis, acute rhinitis, or acute otitis media, both at the time of examination and up to 2 weeks before the examination; (2) patients with infections of the outer ear; (3) patients with tympanic membrane perforation; (4) patients with abnormal ENT anatomy, congenital abnormalities, trauma or malignancy of the ear, nose, and nasopharynx; (5) patients with signs and symptoms of allergic rhinitis.

Laryngopharyngeal reflux was the independent variable and otitis media with effusion was the dependent variable. Laryngopharyngeal reflux variable was determined by calculating the reflux symptom index (RSI) and reflux finding score (RFS). Laryngopharyngeal reflux was diagnosed when RSI > 13 and RFS > 7 [6]. Subjects with RSI score less than 13 and RFS score less than 7 are categorized as non-LPR patient. Otitis media with effusion was determined by ear complaints plus pneumatic otoscopy and tympanometry investigations. Otitis media with effusion was diagnosed if there were one or more ear complaints (intermittent mild ear pain, fullness in the ear, tinnitus, and hearing loss) in one ear or both ears and if the movement of the tympanic membrane was minimal or obstructed in the inspection of otoscopy pneumatic and or tympanogram B conducted in the tympanometry investigation. The prevalence ratio was calculated by dividing the prevalence of OME in LPR group by the prevalence of OME in non-LPR group.

## 3. Study Result and Discussion

The number of samples in this study was 56 subjects. Characteristics of the study subjects are shown in Table 1.

Based on age there was significant difference between LPR and non-LPR groups with p-value of 0.007. LPR happened mostly between the ages of 20 to 40 years, generally an active and productive population group, therefore more susceptible to stress condition and consequently to LPR [7].

There were more males than females in both groups: 57.14% in LPR group and 71.43% in non-LPR group, but there was no significant difference between the two groups (p=0.202). This result has shown difference compared to research by Barbosa et al. at Manaus in 2008 which reported that, based on gender, LPR happened more frequently in females. Barbosa said that the fact that women in the city of Manaus have been under double work shift (sometimes triple) made them more susceptible to LPR, a type of disease that is related to daily stress routine [7].

Most throat complaint was a sense of a lump in the throat either in LPR group or in non-LPR group, by 53.57% in LPR group and 50% in non-LPR group, and there was no significant difference between the two groups with p-value of 0.612. LPR symptoms varied with the most frequent complaints being hoarseness, globus pharyngeus, dysphagia, coughing, throat clearing, and sore throat [5].

Based on the onset of throat complaint obtained 6 months in LPR group and 3 months in non-LPR group, there was significant difference between the two groups with p-value of 0.006. In research by Barbosa et al., the onset of LPR ranged between 1.1 and 5 years. The duration of these complaints shows that the LPR is a chronic condition [7].

The ear complaints in LPR group were mostly fullness in the ear by 25%, followed by 21.43% hearing loss and tinnitus of 10.71%, whereas in non-LPR group the complaints were mostly of tinnitus by 10.71% and hearing loss of 3.57%.

There was significant difference between the two groups with p-value of 0.002, with more ear complaints in LPR group than in non-LPR group. OME symptoms are intermittent mild ear pain, fullness in the ear, and hearing loss [8]. Research of Bargava et al. (2015) found 74% of GERD patients with complaints of fullness in the ear [9].

The main outcome of this study is the ratio prevalence of OME in LPR group. The prevalence of OME in LPR group and in non-LPR group can be seen in Table 2.

Based on the ear complaints, inspection of pneumatic otoscopy and tympanogram B revealed OME in 9 subjects (32.14%) in the LPR group and 2 subjects (7.14%) in the non-LPR group, and there was significant difference between the two groups with p-value of 0.02 [10].

The prevalence ratio was calculated by dividing the prevalence of OME in LPR group by the prevalence of OME in non-LPR group. In this study, the prevalence ratio of OME in LPR group compared to OME in non-LPR group was 4.5. The 95% confidence interval ranges from 1.066 to 18.990, so it can be deduced that laryngopharyngeal reflux is a risk factor of OME. The result also showed that a patient with LPR had 4,5 times greater chance of getting OME than a non-LPR patient [10].

Old age and male gender are factors that contribute significantly to high occurrence of acid exposure to the esophagus. The reflux contents can easily get into the middle ear in old age, which causes auditory tube dysfunction so that the ventilation of the middle ear is disturbed. Exposure to reflux in the long term without adequate treatment will increase the occurrence of OME [10–12].

## 4. Conclusion

The prevalence ratio of otitis media with effusion in laryngopharyngeal reflux group is 4.5 times compared to non-laryngopharyngeal reflux group.

## Figures and Tables

**Table 1:** Characteristics of the study subjects.

Characteristics	LPR (+)	LPR (-)	*p-value*
Age (years)	40(19-72)	30 (19-78)	0.007^a^
Sex			
Male	16(57.14%)	20 (71.43%)	0.202^b^
Female	12(42.86%)	8 (28.57%)	
Throat complaints			
Sore throat	0	1 (3.57%)	0.621^b^
Sense of a lump in the throat	15(53.57%)	14 (50%)	
Hoarseness	6(21.43%)	3 (10.71%)	
Difficulty swallowing	1(3.57%)	1 (3.57%)	
Feels there is mucus in the throat	6(21.43%)	9 (39.29%)	
Onset (months)	6(1-60)	3 (1-24)	0.006^a^
Ear complaints			
Hearing loss	6(21.43%)	1 (3.57%)	0.002^b^
Tinnitus	3(10.71%)	3 (10.71%)	
Fullness in the ear	7(25%)	0	

Information: ^a^Mann-Whitney test; ^b^chi-square test.

**Table 2:** The prevalence of OME in LPR group and in non-LPR group.

	OME (+)		OME (-)		Total		*p-value*	CI 95% (min-max)
	N	%	N	%	N	%		
LPR (+)	9	32.14	19	67.86	28	50	0.02^*∗*^	4.5 (1.066-18.990)
LPR (-)	2	7.14	26	92.86	28	50		
Total	11	19.64	45	80.36	56	100		

Information: ^*∗*^Fisher's exact test.

## Data Availability

The Excel data used to support the findings of this study are available from the corresponding author upon request.

## References

[1] Mills R., Hathorn I. (2016). Aetiology and pathology of otitis media with effusion in adult life. *The Journal of Laryngology & Otology*.

[2] Casselbrant M. L., Mandel E. M., Mandel E. M., Rosen C. A. (2014). *Otitis Media in the Age of Antimicrobial Resistance dalam Bailey’s Head & Neck Surgery-Otolaryngology*.

[3] Roozbahany N. A., Majdinasab N., Oroei M., Nateghinia S. (2016). Adult Onset Otitis Media with Effusion: Prevalence and Etiology. *Journal of Hearing Science Otolaryngology*.

[4] Lipan M. J., Reidenberg J. S., Laitman J. T. (2006). Anatomy of reflux: A growing health problem affecting structures of the head and neck. *Anatomical Record - Part B New Anatomist*.

[5] Koufman J. A., Aviv J. E., Casiano R. R., Shaw G. Y. (2002). Laryngopharyngeal reflux: position statement of the committee on speech, voice, and swallowing disorders of the american academy of otolaryngology-head and neck surgery. *Otolaryngology—Head and Neck Surgery*.

[6] Belafsky P. C., Postma G. N., Koufman J. A. (2001). The validity and reliability of the reflux finding score (RFS). *The Laryngoscope*.

[7] Barbosa A. B., Berberena L. S., Barbosa K. L. P., Ribeiro D. S. (2008). The Laryngeal Manifestations of Laryngeal-pharynx Reflux and its Correlation with the Population of Manaus City. *International Archives of Otorhinolaryngology*.

[8] Rosenfeld R. M., Culpepper L., Doyle K. J. (2016). Clinical Practice Guideline: Otitis Media with Effusion. *Otolaryngology—Head and Neck Surgery*.

[9] Bhargava A., Cherian M., Cherian T. A., Gupta S., Murthy P. (2015). Gastroesophageal Reflux Disease in Patients with Eustachian Tube Catarrh. *International Journal of Phonosurgery & Laryngology*.

[10] Karyanta M., Sastrowiyoto S., Wulandari D. P. (2017). Rasio Prevalensi Otitis Media Efusi pada Refluks. *Laringofaring*.

[11] Sone M., Katayama N., Kato T. (2012). Prevalence of laryngopharyngeal reflux symptoms: Comparison between health checkup examinees and patients with otitis media. *Otolaryngology - Head and Neck Surgery (United States)*.

[12] Brunworth J. D., Mahboubi H., Garg R., Johnson B., Brandon B., Djalilian H. R. (2014). Nasopharyngeal Acid Reflux and Eustachian Tube Dysfunction in Adults. *Annals of Otology, Rhinology & Laryngology*.

